# Solving another piece of the thrombotic thrombocytopenic purpura puzzle?

**DOI:** 10.1016/j.rpth.2026.103429

**Published:** 2026-03-26

**Authors:** Paschalis Evangelidis, Eleni Gavriilaki

**Affiliations:** 1 Second Propedeutic Department of Internal Medicine, Hippokration Hospital, Aristotle University of Thessaloniki, Thessaloniki, Greece; 2 Endothelial Injury Excellence Centre, Hematology Department, Bone Marrow Transplantation Unit, G. Papanikolaou General Hospital of Thessaloniki, Thessaloniki, Greece

Immune-mediated thrombotic thrombocytopenic purpura (iTTP) is a rare, life-threatening hematological disorder characterized by the presence of microangiopathic hemolytic anemia, thrombocytopenia, and target organ damage due to microvascular thrombosis, which leads to tissue ischemia [1]. In iTTP, which is an autoimmune disorder, autoantibodies can recognize and bind to specific regions of a disintegrin and metalloproteinase with a thrombospondin type 1 motif, member 13 (ADAMTS-13), thereby inhibiting the enzyme [2]. Subsequently, ultralarge von Willebrand factor multimers accumulate in the endothelium of small vessels, thereby increasing platelet adhesion under circumstances of high shear stress [2]. Before the universal use of therapeutic plasma exchange and immunosuppression in the management of patients with iTTP, mortality rates exceeded 90% in untreated cohorts [3]. Following the introduction of therapeutic plasma exchange, corticosteroids, rituximab, and, more recently, targeted therapies, such as caplacizumab, mortality in the acute setting has been reduced to <5% to 10% [[4], [5], [6]].

Despite the achievement of improved survival outcomes for individuals living with iTTP, a significant percentage of them (up to 50%, as reported in some studies) experience relapses after the first disease episode [7]. As expected, persistent severe ADAMTS-13 deficiency (<10%-20%) during remission has been correlated with increased risk for relapse [1]. Pre-emptive administration of rituximab in subjects with iTTP during remission with severe ADAMTS-13 deficiency has significantly reduced relapse rates by up to 85%, and in some cohorts to zero [8]. Nevertheless, recovery of ADAMTS-13 activity is not durable in all patients, with a substantial number requiring repeated treatment cycles to achieve sustained ADAMTS-13 activity [8].

Moreover, long-term iTTP survivors experience several complications and chronic conditions, such as hypertension, major cardiovascular events (mainly acute ischemic stroke), neurocognitive dysfunction, chronic kidney disease, and reduced quality of life [9,10]. Importantly, women living with iTTP face a high risk for disease relapse and maternal–fetal complications in subsequent pregnancies [11,12]. Persistent ADAMTS-13 deficiency during remission disrupts the physiological balance between ADAMTS-13 and von Willebrand factor, thus promoting platelet activation, endothelial injury, and impaired microvascular flow [13]. Clinical and experimental data have shown an association between reduced ADAMTS-13 activity and microvascular dysfunction, as well as an increased risk of cerebrovascular and cardiovascular events, even independently of traditional risk factors [14]. In iTTP survivors, this chronic prothrombotic and microvascular injury state might contribute to cumulative vascular damage, manifesting as increased rates of the aforementioned complications and conditions [14].

A major challenge in the management of iTTP is that, despite current therapies effectively controlling acute episodes and significantly improving survival, immune tolerance to ADAMTS-13 is not restored [3]. Underlying autoimmune mechanisms, including recurrent or persistent production of autoantibodies against ADAMTS-13, remain unresolved in some patients [15]. CD20-negative plasma cells might be implicated in this chronic production of autoantibodies [15].

Thus, although available treatment paradigms effectively control acute episodes and improve survival by suppressing anti–ADAMTS-13 autoantibody production, they do not restore immune tolerance to ADAMTS-13, resulting in persistence or recurrence of autoimmunity in some cases after withdrawal of immunosuppressive therapy. Given this limitation, there is a need to develop precise, antigen-specific strategies that can selectively target the pathogenic immune response while preserving overall immune function. A potential solution to this problem is the restoration of peripheral tolerance to ADAMTS-13, which would represent a shift toward addressing the root cause of iTTP.

Maintenance of immune tolerance is achieved by two mechanisms: central, which involves the elimination of self-reactive lymphocytes during their development, and peripheral, in which autoreactive cells that have escaped central mechanisms are controlled [16]. Given that central tolerance cannot be reestablished in adulthood, peripheral tolerance plays a pivotal role in the pathogenesis of adult-onset autoimmune diseases, such as iTTP, by restraining potentially pathogenic immune responses through various mechanisms, including anergy, deletion, regulatory T cells, and inhibitory signaling pathways [16]. Furthermore, it is well established that the outcome of peripheral tolerance largely depends on antigen context, as antigens encountered in noninflammatory (silent) settings favor immune inactivation rather than activation [16]. For instance, physiological clearance of apoptotic cells and red blood cells (RBCs) constitutes a prototype of a silent pathway, promoting tolerance by facilitating antigen presentation without the need for costimulation by proinflammatory factors and, thus, protecting immune homeostasis. Specifically, erythrophagocytosis is a physiological process in which damaged RBCs are destroyed by macrophages in a noninflammatory, immune-silent way. Additionally, RBCs are characterized by a lack of antigen-presenting capacity, given that antigens coming from their clearance are presented by professional antigen-presenting cells in the absence of costimulatory and inflammatory signals [17]. Therefore, the recognition of RBC-derived antigens by T cells favors functional inactivation or differentiation into regulatory T cells over immune activation [17]. It has been shown that delivery of antigens via RBCs can exploit the above-described mechanisms to selectively suppress T cell antigen-specific responses and thus promote durable peripheral tolerance [17]. This can provide a biologically rational and targeted alternative to systemic immunosuppressive therapies.

Given the increased risk for relapse episodes and the burden of complications that long-term survivors of iTTP experience, along with the potential of peripheral tolerance-inducing therapies in autoimmune disorders, in this issue of *Research and Practice in Thrombosis and Haemostasis*, Dierickx et al. [18] provided, for the first time to our knowledge, an important advance in this field. Specifically, they developed a biologically rational strategy for the controlled and physiological delivery of ADAMTS-13–derived T cell epitopes, aiming to achieve antigen-specific tolerance in iTTP. Understanding the above-described mechanism of antigen delivery via RBCs and the fundamental homeostatic pathway of RBC clearance, their objective was to introduce an ADAMTS-13 antigen (FINVAPHAR amino acid sequence in the CUB domain of ADAMTS-13) to the immune system in circumstances of immune silence in order to induce mechanisms associated with peripheral immune tolerance and not the activation of inflammatory responses (Figure).

**Figure:**
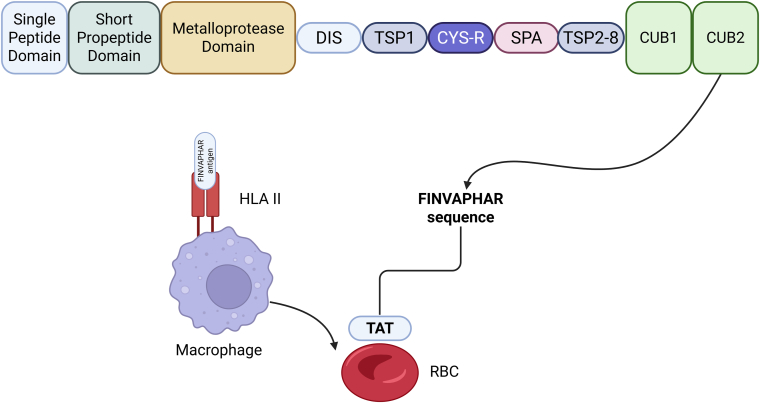
RBC-mediated delivery of an ADAMTS-13–derived T cell epitope through immune-silent erythrophagocytosis. ADAMTS-13, a disintegrin and metalloproteinase with a thrombospondin type 1 motif, member 13; HLA, human leukocyte antigen; RBC, red blood cell; TAT, transactivator of transcription; CUB, complement C1r/C1s, Uegf, Bmp1; CYS-R, cysteine-rich; DIS, disintegrin-like; SPA, spacer; TSP, thrombospondin type 1 repeat. Created in BioRender. Evangelidis, P. (2026) https://BioRender.com/fmpium1.

Therefore, using the transactivator of transcription protein of HIV as a fusion molecule, it was reported that FINVAPHAR (ADAMTS-13 peptides) can be loaded on the surface of RBCs in a concentration-dependent manner and then taken up by macrophages via the process of erythrophagocytosis. The uptake of the ADAMTS-13 antigen conjugated with transactivator of transcription on the surface of RBCs by macrophages leads to direct presentation of FINVAPHAR-containing peptides on human leukocyte antigen (HLA) class II molecules, as it was shown by high-resolution immunopeptidomic analysis. HLA class II molecules can present antigens on CD4^+^ T cells (autoreactive), which, upon their activation, can further promote the differentiation of autoreactive B cells to plasma cells, which will be able to produce autoantibodies implicated in iTTP pathogenesis [16]. Peripheral tolerance to ADAMTS-13–derived antigens (eg, FINVAPHAR) may suppress this process.

An important insight in this study includes the HLA restriction observed during this approach. Presentation of ADAMTS-13–derived antigens was more robust in macrophages collected from donors carrying a well-established iTTP genetic risk allele in the European population (HLA-DRB1∗11). At the same time, limited antigen presentation across other HLA haplotypes may indicate either the specificity or the possibility of modulation of the proposed methodology.

We would like to highlight that this study did not provide direct evidence of peripheral immune tolerance but introduced important essential elements for the induction of this phenomenon, such as controlled delivery of antigens, physiological uptake by macrophages, and iTTP-relevant HLA class II presentation. This link between antigen exposure and a naturally occurring clearance pathway offers a connection to theoretical concepts concerning immune tolerance with a clinically relevant methodology. Furthermore, RBCs were underlined as active contributors to the regulation of the immune system, adding also significant knowledge to our understanding of immune tolerance implicated mechanisms in iTTP.

The aforementioned approach may be used during the remission phase of iTTP, after its safety and efficacy have been investigated in experimental and subsequent clinical studies, and not during acute disease episodes, where rapid and aggressive control of microvascular thrombosis is of paramount importance. Indeed, peripheral tolerance-inducing therapeutics are unlikely to replace the established standard of care in the acute setting but may be considered adjunctive interventions with the objective of relapse prevention and the promotion of durable immune control once clinical remission is achieved. Therefore, RBC-based antigen delivery could complement current immunosuppressive regimens as a solution to the underlying autoimmune response.

Future research agenda questions in this field might include the following:

• Preclinical animal studies in order to investigate the safety and efficacy of this approach.
• Identification of the potential target population. This approach should be evaluated during remission, particularly in patients with persistently low ADAMTS-13 activity who face a greater risk of relapse.
• Precision medicine strategies. Possible integration of ADAMTS-13 activity monitoring with emerging-risk stratification tools, such as HLA genotyping and anti–ADAMTS-13 autoantibodies profiling.
• Therapeutic role. Assessment of this approach as an adjunct rather than a replacement in acute episode management.
• Combination strategies. Exploration of a possible combination between this approach and recombinant ADAMTS-13 (rADAMTS-13), which emerges as a potential therapeutic option in iTTP.

Finally, in the 2025 focused update of the 2020 International Society on Thrombosis and Haemostasis guidelines for the management of thrombotic thrombocytopenic purpura, rADAMTS-13 is now included in the management of individuals with congenital thrombotic thrombocytopenic purpura [19]. Sakai et al. [20] recently reported the emergence of anti–ADAMTS-13 alloantibodies in a patient receiving rADAMTS-13 replacement therapy [20]. In this clinical scenario, antigen-specific tolerance strategies, such as immune-silent RBC-mediated antigen delivery, could, theoretically, complement enzyme replacement by reducing immunogenicity and preserving long-term therapeutic efficacy.

## References

[1] Sukumar S., Gavriilaki E., Chaturvedi S. (2021). Updates on thrombotic thrombocytopenic purpura: recent developments in pathogenesis, treatment and survivorship. Thromb Update.

[2] Papakonstantinou A., Kalmoukos P., Mpalaska A., Koravou E.E., Gavriilaki E. (2024). ADAMTS13 in the new era of TTP. Int J Mol Sci.

[3] Subhan M., Scully M. (2022). Advances in the management of TTP. Blood Rev.

[4] Coppo P., Bubenheim M., Benhamou Y., Völker L., Brinkkötter P., Kühne L. (2025). Caplacizumab use in immune-mediated thrombotic thrombocytopenic purpura: an international multicentre retrospective cohort study (The Capla 1000+ project). EClinicalMedicine.

[5] Gavriilaki E., Nikolousis E., Koravou E.E., Dimou-Besikli S., Kartsios C., Papakonstantinou A. (2023). Caplacizumab for immune thrombotic thrombocytopenic purpura: real-world multicenter data. Front Med (Lausanne).

[6] Melanis K., Theodorou A., Palaiodimou L., Bakola E., Chondrogianni M., Tsikalakis G. (2026). Caplacizumab as an emerging treatment for patients with thrombotic thrombocytopenic purpura presenting with acute ischemic stroke. Neurol Clin Pract.

[7] Selvakumar S., Liu A., Chaturvedi S. (2023). Immune thrombotic thrombocytopenic purpura: spotlight on long-term outcomes and survivorship. Front Med (Lausanne).

[8] Owattanapanich W., Wongprasert C., Rotchanapanya W., Owattanapanich N., Ruchutrakool T. (2019). Comparison of the long-term remission of rituximab and conventional treatment for acquired thrombotic thrombocytopenic purpura: a systematic review and meta-analysis. Clin Appl Thromb Hemost.

[9] Sukumar S., Brodsky M., Hussain S., Yanek L., Moliterno A., Brodsky R. (2022). Cardiovascular disease is a leading cause of mortality among TTP survivors in clinical remission. Blood Adv.

[10] Yu J., Brown J., Meade J., Gerber G.F., Streiff M.B., Kraus P. (2025). Progressive silent cerebral infarction is associated with stroke and persistent cognitive impairment in survivors of iTTP. Blood Adv.

[11] Mahmoud A.A., Eltaher B., Shah P., Antun A., Masias C., Sridharan M. (2025). Fetal and maternal morbidity in pregnant patients with thrombotic thrombocytopenic purpura: a nationwide analysis. Transfusion.

[12] Gavriilaki E., Tsakiridis I., Kalmoukos P., Papakonstantinou A., Mauridou M., Kotsiou N. (2024). A rare case of thrombotic thrombocytopenic purpura during pregnancy with a successful outcome despite ovarian hyperstimulation syndrome during treatment. Thromb Update.

[13] Sonneveld M.A.H., de Maat M.P.M., Leebeek F.W.G. (2014). Von Willebrand factor and ADAMTS13 in arterial thrombosis: a systematic review and meta-analysis. Blood Rev.

[14] Sukumar S., Mazepa M.A., Chaturvedi S. (2023). Cardiovascular disease and stroke in immune TTP-challenges and opportunities. J Clin Med.

[15] Chen M., Shortt J. (2022). Plasma cell directed therapy for immune thrombotic thrombocytopenic purpura (iTTP). Transfus Med Rev.

[16] Weinstein E., Peeva E., Putterman C., Diamond B. (2004). B-cell biology. Rheum Dis Clin N Am.

[17] Cremel M., Guérin N., Horand F., Banz A., Godfrin Y. (2013). Red blood cells as innovative antigen carrier to induce specific immune tolerance. Int J Pharm.

[18] Dierickx M., Groten S., Miranda M., Langerhorst P., van der Zwaan C., Mul E. (2026). A red blood cell-based antigen delivery system to facilitate T cell epitope presentation to promote peripheral tolerance to ADAMTS13 in immune-mediated TTP. Res Pract Thromb Haemost.

[19] Zheng X.L., Al-Housni Z., Cataland S.R., Coppo P., Geldziler B., Germini F. (2025). 2025 focused update of the 2020 ISTH guidelines for management of thrombotic thrombocytopenic purpura. J Thromb Haemost.

[20] Sakai K., Hamamura A., Tanabe S., Kajita M., Fujimura Y., Matsumoto M. (2026). Alloantibody to recombinant ADAMTS13 reduces clinical efficacy in congenital TTP: an emerging therapeutic concern. Am J Hematol.

